# Bone Defect Regeneration Using Mesenchymal Stem Cells from Bichat’s Fat Pad

**DOI:** 10.17691/stm2026.18.3.04

**Published:** 2026-06-30

**Authors:** S.V. Ostapets, O.S. Kosareva, M.N. Drovosekov, E.V. Nikitenko, M.A. Surovtseva, I.I. Kim, O.V. Poveshchenko

**Affiliations:** 1 Assistant, Department of Surgical Dentistry, Dental Implantation, and Maxillofacial Surgery, Faculty of Dentistry; Novosibirsk State Medical University, 52 Krasny Prospekt, Novosibirsk, 630091, Russia; 2 Assistant, Department of Orthopedic Dentistry, Faculty of Dentistry; Novosibirsk State Medical University, 52 Krasny Prospekt, Novosibirsk, 630091, Russia; 3 MD, DSc, Associate Professor, Head of the Department of Surgical Dentistry, Dental Implantation, and Maxillofacial Surgery, Faculty of Dentistry; Novosibirsk State Medical University, 52 Krasny Prospekt, Novosibirsk, 630091, Russia; 4 MD, PhD, Pathologist, Department of Clinical Pathomorphology; City Clinical Hospital No.1, 6 Zalesskogo St., Novosibirsk, 630047, Russia; 5 MD, PhD, Leading Researcher, Laboratory of Cell Technologies; Research Institute of Clinical and Experimental Lymрhology — Branch of the Institute of Cytology and Genetics, Siberian Branch of Russian Academy of Sciences, 2 Timakova St., Novosibirsk, 630060, Russia; 6 MD, PhD, Senior Researcher, Laboratory of Cell Technologies; Research Institute of Clinical and Experimental Lymрhology — Branch of the Institute of Cytology and Genetics, Siberian Branch of Russian Academy of Sciences, 2 Timakova St., Novosibirsk, 630060, Russia; 7 MD, DSc, Head of the Laboratory of Cellular Technologies; Research Institute of Clinical and Experimental Lymрhology — Branch of the Institute of Cytology and Genetics, Siberian Branch of Russian Academy of Sciences, 2 Timakova St., Novosibirsk, 630060, Russia

**Keywords:** mesenchymal stem cells, Bichat’s fat pad, repair of bone defect, regeneration

## Abstract

**The aim of the study** was to assess the efficiency of the mandibular bone defect regeneration by administering mesenchymal stem cells (MSC) from the Bichat’s fat pad (B-MSC), as well as the osteoblasts differentiated from them, according to the morphological findings.

**Materials and Methods:**

The experimental study was carried out on male Wistar rats (n=120) aged 2 months, weighing 250–300 g. B-MSCs and the osteoblasts differentiated from them were used for the regeneration of rat mandibular bone defects formed by a round bur. In the control group, the defect was not filled, the defect was healing under the blood clot. The biopsy specimens were histologically examined on days 14, 30, and 90 after the injury formed.

**Results:**

The infiltration of the bone defect site by inflammatory cells was revealed for all study groups 14 days after the injury. The groups with cell administration showed the resorption of bone trabeculae (B-MSC and osteoblast groups) and a large number of capillaries (an osteoblast group). On day 30 after the injury, the preparations of the groups with cell administration demonstrated a large number of capillaries (B-MSC and osteoblast groups) and primitive bone trabeculae with osteoblasts (an osteoblast group). Active osteoclastic resorption and inflammatory infiltration were revealed in the control group. The groups with cell administration 90 days after the injury, in contrast to the control group, were found to have the formation of spongy mature bone tissue (B-MSC and osteoblast groups), the presence of bone mineralization and myeloid bone marrow (an osteoblast group) in the bone defect zone. It indicated high regenerative potential of the cells used when replacing a bone defect and the significant reduction of a regeneration period.

## Introduction

Currently, one of the most urgent problems of modern dentistry is the repair of bone tissue defects in the upper and lower jaws and the functional and esthetic parameters of dentofacial system using dental implants in case of tooth loss. The placement of endosseous implants enables to construct fixed dentures regardless of the number of missing teeth. Annually, over 9 million implants are being placed worldwide [1].

The prosthetic efficiency of implant prosthodontics depends significantly on bone tissue volume and quality in the area of planning implantation, since surgeons frequently face the problem of bone deficiency [2–5]. In dentistry, bone tissue repair using autografts is a gold standard. At present, autologous bone can be considered the optimal material for transplantation since it exhibits, at the same time, osteoconductive, osteoinductive, and osteogenic properties. Additionally, its significant advantages are the absence of immunologic responses and the complete transformation of an autologous graft into the surrounding bone tissue [6]. And it should also be noted that the repair of the maxillary alveolar process defect and the mandibular alveolar part by autogenous bone material has a number of disadvantages, which include: an increase of surgery time; supplementary traumatization due to a graft taken from the donor’s zone, which is occasionally accompanied by the formation of hematomas and their infection and neurovascular bundle injury; the discrepancy between the autograft taking and the defect size of the recipient’s zone [7, 8].

In recent years, there have been the reports on the data on the efficiency of mesenchymal stem cells (MSC) in bone repair. MSCs are able to differentiate in different directions and exhibit high osteogenic potential [9, 10]. By now, MSCs from different sources have been obtained and characterized [11, 12], and MSCs isolated from adipose tissue are the most common cells used in regenerative medicine [12]. In contrast to bone marrow, adipose tissue has a number of advantages consisting in the simpler way of tissue taking and the great number of cells isolated from the unit volume of the taken material [13]. Oral adipose tissue (buccal fat pads, or Bichat’s fat pads (B-MSC)) can serve as an accessible source of MSCs in dentistry and osteofacial surgery. Previously, we showed MSCs isolated from buccal fat pads to have higher proliferative activity and osteogenic differentiation potency compared with MSCs from bone marrow and visceral fat [14].

**The aim of the study** was to assess the efficiency of the mandibular bone defect regeneration by administering mesenchymal stem cells from the Bichat’s fat pad, as well as the osteoblasts differentiated from them, according to the morphological findings.

## Materials and Methods

MSCs (B-MSCs) were isolated from the adipose tissue of the Bichat’s pad, surgically removed in the dental clinic, RecomenDent (Russia). All patients (n=6) signed the informed consent for surgery, tissue retrieval, and being involved in research. The study was approved by the Ethics Committee of Research Institute of Clinical and Experimental Lymphology (Russia) (protocol No.7 dated October 10, 2007). The taken adipose tissue was twice washed in buffered saline followed by crushing using a scalpel, and then it was treated with 0.05% collagenase solution (*Clostridium histolyticum*, type I; Sigma-Aldrich, USA) with 2% fetal bovine serum added (FBS; HyClone, USA) at 37°С within 18–20 h. The isolated cells were cultured in complete growth medium consisting of DMEM (BioloT, Russia) with the addition of 10% FВS (HyClone, USA), 2 mM L-glutamine (BioloT, Russia), 40 μkg/ml gentamycine (Dalchimpharm, Russia) at 37°C and 5% CO_2_ renewing the medium every 3–4 days. The cells were removed at passage using trypsin-versene solution (1:1) (BioloT, Russia).

The directed osteogenic differentiation of 3–5-passage B-MSCs was performed in the osteoinduction medium consisting of DMEM with the addition of 10% FBS, 40 μkg/ ml gentamycine, 50 μkg/ml of ascorbic acid (Sigma-Aldrich, USA), 5·10^–7^ М dexamethasone (Shreya Life Sciences, India) and 10 mM sodium glycerophosphate (Sigma-Aldrich, USA) within 21 days [15]. The medium was changed every 3–4 days. Calcium salt accumulation was confirmed by alizarin red staining (Sigma-Aldrich, USA). The cells were removed using trypsin-versene solution (1:1) (BioloT, Russia), calculated and administered to the animals at the rate of 1·10^6^ cells per animal. The cell morphology was studied using Axio Observer microscope (ZEISS, Germany).

The experimental study was carried out on 120 male Wistar rats aged 2 months, weighing 250–300 g. All procedures with animals were approved by the Ethics Committee of Novosibirsk State Medical University (Russia) (protocol No.163 dated December 18, 2024) and carried out in accordance with the European Convention for the Protection of Vertebrate Animals used for Experimental and Other Scientific Purposes (Strasbourg, 1986). Under general anesthesia (by an intramuscular injection of 0.1 ml Telazol; Zoetis Inc, USA) the left submandibular space was incised exposing the mastication muscle followed by dissecting away the tendon of the mastication muscle from the angle of the mandible, and then the angle of the mandible was skeletonized. Perforated trepanation of the mandible was performed using a round bur under water cooling. The trephine opening diameter was 1 mm.

The animals were divided into 3 groups (40 rats in each group): B-MSC administration; administration of osteoblasts; injury (no therapy, control). A formed bone defect was filled with cells (B-MSCs or osteoblasts) at the rate of 1·10^6^ cells per animal. The cells were preliminary absorbed on a collagen sponge (LLC “Luzhsky Plant “BELKOZIN”, Russia) to prevent them from diffluence. In the controls the formed defect was not filled, the defect was healing under the blood clot. The wound was sutured layer-by-layer in all experimental groups. To assess the response to the treatment, the animals were withdrawn from the experiment on days 14, 30, and 90 after the defect formation.

The mandibular fragments, about 0.5 cm^2^ in area, containing the bone defect, were taken for histological examination. The samples under study were fixed in 10% neutral formalin solution for 48 h. The bone fragments were isolated and made free from soft tissues, and then exposed to decalcification in the EDTA-based decalcifying solution SoftiDec (LLC “ErgoProduction”, Russia). The tissue samples were brought through high-strength alcohol and embedded in paraffin. Longitudinal and transverse regenerated sections were made from the paraffin blocks. The sections 5–6 μm thick were made using the rotary microtome Leica RM 2555 (Leica Biosystems/Microsystems, Germany) and were hematoxylin and eosin stained. The histology specimens were studied, and the microphotographs were taken using the optic digital system AxioPlan 2 Imaging (ZEISS, Germany).

## Results

**Figure 1.**
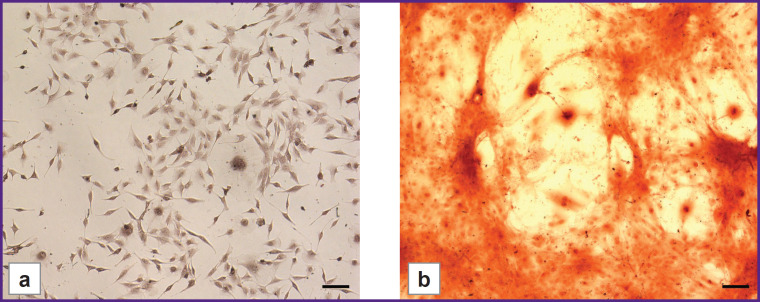
Mesenchymal stem cells obtained from Bichat’s fat pad, prior to osteodifferentiation (a) and in 21 days of culturing in the osteogenic medium (b) Alizarin red staining; magnification 100×; bar — 100 μm

Previously, we had succeeded in obtaining and characterizing MSCs from buccal fat pads. B-MSCs were shown to have the phenotype of CD90^+^/CD73^+^/ CD105^+^/CD34^–^/CD45^–^; they exhibited the ability to differentiate in adipogenic and osteogenic directions [14, 16]. Figure 1 represents the morphology of the cells used in the experiment. B-MSCs were fusiform (Figure 1 (a)). The osteoblasts resulted from differentiation were large cuboid or polygonal cells with basophilic cytoplasm. The osteoblasts produced the matrix containing calcium salts, which were revealed by a specific stain — alizarin red (Figure 1 (b)).

**Figure 2.**
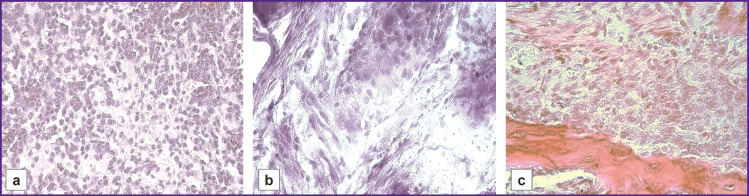
Mandibular defect area 14 days after formation: (a) control group (no defect filling); (b) the defect filling with mesenchymal stem cells isolated from Bichat’s fat pad; (c) the defect filling with osteoblasts. Hematoxylin and eosin staining; magnification 400×

The histological examination on day 14 after the defect formation in the control group showed the bone defect area to be filled with granulation tissue with marked diffuse polymorphocellular inflammatory infiltration (Figure 2 (a)). There was revealed the presence of rare paretically dilated capillaries with swollen endothelial cells.

On day 14 after the defect formation and B-MSC administration (Figure 2 (b)) the bone defect area was filled with the granulation tissue with diffuse polymorphocellular inflammatory infiltration. In some sections there was a formed capsule around the defect. The capsule contained loose fibrous connective tissue with small oxyphil areas of necrotic bone tissue and the resorption (rarefication) foci of trabeculae, with slight infiltration by neutrophils. It must be the response to a direct mechanic bone injury when forming a defect; and it was also due to the blood flow peculiarities in bone tissue that determined the mediated destruction of the ischemic bone adjacent to the wound site. The adjacent bone tissue surrounding the defect had significantly marked proliferation of mesenchymal cell elements.

In the group with a defect substituted by the osteoblasts differentiated from B-MSC, on day 14 after injury (Figure 2 (c)) the modeled bone defect area was filled with granulation tissue with diffuse polymorphocellular inflammatory infiltration, where small fragments of partially reabsorbed trabeculae could be identified. In the periosteal region there were revealed slight lymphohistiocytic infiltration with a small quantity of neutrophils and numerous capillaries with swollen endothelial cells.

**Figure 3.**
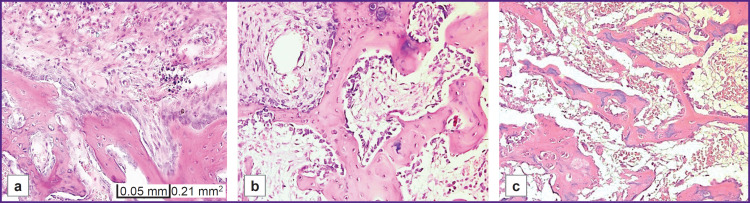
Mandibular defect area following 30 days after formation: (a) control group (no defect filling); (b) the defect filling with mesenchymal stem cells isolated from Bichat’s fat pad; (c) the defect filling with osteoblasts. Hematoxylin and eosin staining; magnification 200×

On day 30 after injury the bone defect area in the control group was filled with osteogenic reticular tissue with mild osteoblastic response and the margins of “tender” osteoid (Figure 3 (a)). In the fibroreticular tissue there were rare small capillary-type vessels. There was observed active osteoclastic resorption in the area of change-over structures. It should be noted that the inflammation was productive, as part of the infiltrate there were predominantly found macrophages, lymphocytes, and plasmatic cells. There was a small quantity of neutrophils in the infiltrate.

The modeled bone defect area filled with B-MSCs contained osteogenic fibroreticular tissue with forming trabeculae surrounded by osteoblasts (Figure 3 (b)). In the surrounding fibroreticular tissue there were numerous capillaries and focal lymphohysteocytic infiltration.

30 days after the bone defect formation and its substitution with osteoblasts, the defect was filled with osteogenic fibroreticular tissue with the margins of “tender” osteoid and single primitive trabeculae. The trabeculae of irregular organization were disorderly arranged. There was marked osteoblastic reaction around the trabeculae. The surrounding fibroreticular tissue contained numerous capillaries (Figure 3 (c)).

**Figure 4.**
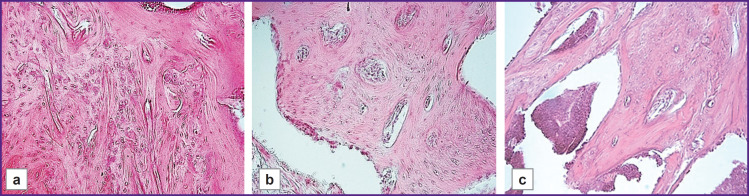
Mandibular defect area following 90 days after injury: (a) control group (no defect filling); (b) the defect filling with mesenchymal stem cells isolated from Bichat’s fat pad; (c) the defect filling with osteoblasts. Hematoxylin and eosin staining; magnification 200×

In the control group, on day 90 of the experiment, the former defect area had formed woven bone; however, in the center there were still the regions with change-over trabeculae with the traces of their absorption by osteoclasts (Figure 4 (a)).

On day 90 after injury and B-MSC administration, the modeled defect area had the formed tissue containing spongy mature bone tissue typical for this region. In the intertrabecular spaces there was mature fibroreticular tissue with single osteoclasts. There was no myeloid bone marrow in the intertrabecular spaces (Figure 4 (b)).

By the end of day 90 of the experiment, the defect area substituted by osteoblasts had the formed tissue consisting of spongy mature bone tissue. The central regions were found to have change-over slightly calcified trabeculae. In the intertrabecular spaces there was the myeloid bone marrow (Figure 4 (c)).

## Discussion

MSC efficiency in osteoinduction and osteogenesis was demonstrated in *in vitro* and *in vivo* studies [17–19]. MSCs under the action of specific soluble microenvironmental factors are known to be able to differentiate into osteoblasts, adipocytes, and chondrocytes [12, 20]. MSCs provide paracrine effects due to the secretion of growth factors and cytokines. They exhibit profound immune modulating properties. MSCs produce anti-inflammatory factors, such as nitrogen oxide, indoleamine 2,3-dioxygenase, as well as anti-inflammatory cytokines (IL-10 and IL-12p40) and chemokines; suppress the production of inflammatory cytokines IL-6, TNF-α, and IFN-γ [21].

Our findings suggest that on day 14 after the bone injury all experimental groups had similar histological pattern related to the infiltration of the defect by inflammatory cells (see Figure 2). However, the groups with administration of cells (B-MSC or osteoblasts) were found to have the resorption of trabeculae and a great number of capillaries (the group with osteoblasts administered), it suggesting the bone regeneration acceleration at this period (see Figure 2 (b, c)). A bone injury is accompanied by local and systemic release of anti-inflammatory cytokines. It attracts the immune cells secreting inflammatory mediators to the wound site. The mediators (e.g., TNF-α), and also TGF-β1 released by the bone matrix, and chemokines, such as SDF-1 (stromal cell-derived factor-1) produced by the injured bone cells cause endogenic involvement of MSCs into the injury site [20]. Endogenic or exogenic MSC migration to the bone injury site is the first step in bone regeneration [22].

On day 30 after the injury, all preparations in the bone defect site were revealed to have osteogenesis activation with forming the margins of “tender” osteoid and single primitive random trabeculae (see Figure 3). It should be noted that in the control group (no administration of cells) there are still the active osteoclastic bone resorption and the moderate inflammatory reaction in the injury site (see Figure 3 (a)). The groups with cell administration were characterized by a great number of capillaries (see Figure 3 (b, c)), and the group with osteoblasts administered — by primitive trabeculae with osteoblasts inside (see Figure 3 (c)). Transplanted MSCs were shown to be able to promote bone regeneration also due to angiogenesis stimulation. MSCs when being in hypoxic perivascular niches were expressing HIF-1α in response to hypoxic condition in the injury site, and they induced the expression of angiogenic factors, such as VEGF, TGF-β, SDF-1 contributing to vascular invasion [19].

On day 90 after the injury, the bone defect regeneration in the control group was not completed yet, as evidenced by the presence of woven bone and the areas of resorption by osteoclasts (see Figure 4 (a)). In contrast to the control group, in the groups with cell administration, on day 90 after the injury, the bone defect was filled with mature spongy bone tissue, and the group with osteoblasts administered was found to have the margins of the slightly mineralized organic bone matrix around osteoblasts and myeloid bone marrow in the intertrabecular spaces (see Figure 4 (b, c)). MSCs during bone remodeling are known to differentiate into osteoblasts providing bone formation [23]. The primary therapeutic effect of MSCs is related to their incorporation to the host’s tissues, and also to their ability to osteodifferentiate, however, there is the paracrine effect on endogenic MSCs due to the secretion of growth factors and cytokines (VEGF, TGF-β, IL-1β, IL-6, IL-8) that finally results in accelerating bone regeneration. MSCs differentiating into osteoblasts were shown to be regulated by a signal pathway Wnt/ β-catenin. β-catenin participates in supporting the differentiation of MSC precursors into mature osteoblasts increasing the regulation of osteogenic regulators Runx2, Dlx5, and Osterix [24]. MSCs can express BMP-2 in an injury site causing the differentiation of these cells into osteoblasts due to autocrine activation of Smad and MAPK (mitogen-activated protein kinase pathway) pathways. BMP-2 plays a significant role in bone healing due to the involvement into new bone tissue formation, the increase of osteoblastic functions and keeping the dynamic balance of the neoformed bone tissue [25].

The present study findings demonstrated the reduction of bone regeneration period in filling a bone defect with osteoblasts. A mineralization process was shown to have two stages: the first is related to the formation of vesicles in the cytoplasm of osteoblasts containing hydroxyl apatites, bone proteins and enzymes; the second stage proceeds in the matrix with the involvement of vesicles, osteoblasts separated from membranes, type I collagen and other components synthesized by osteoblasts [26]. Therefore, the observed in the present study reduction of bone regeneration period when administering osteoblasts in a bone defect can be associated with a preliminary procedure of an *in vitro* MSC differentiation into osteoblasts and, consequently, the first mineralization stage proceeding in the osteoblastic cytoplasm.

## Conclusion

The experimental findings showed the formation of mature spongy bone tissue in the rat mandibular defect 90 days after administering mesenchymal stem cells from Bichat’s fat pad and the osteoblasts differentiated from them, in contrast to the controls (the group with no cell administration). It enables to conclude the high regenerative potential of the used cells when substituting the bone defect and the significant reduction of regeneration time.
